# Honey Bee Colonies Remote Monitoring System

**DOI:** 10.3390/s17010055

**Published:** 2016-12-29

**Authors:** Sergio Gil-Lebrero, Francisco Javier Quiles-Latorre, Manuel Ortiz-López, Víctor Sánchez-Ruiz, Victoria Gámiz-López, Juan Jesús Luna-Rodríguez

**Affiliations:** 1 Department of Zoology, University of Córdoba, Córdoba 14071, Spain; ilenro89@gmail.com (S.G.-L.); victoriagamizlopez@gmail.com (V.G.-L.); 2 Department of Computer Architecture, Electronics and Electronic Technology, University of Córdoba, Córdoba 14071, Spain; el1qulaf@uco.es (F.J.Q.-L.); v644452553@gmail.com (V.S.-R.); el1luroj@uco.es (J.J.L.-R.)

**Keywords:** precision beekeeping, precision apiculture, bee colony monitoring, wireless sensor network, internet of things

## Abstract

Bees are very important for terrestrial ecosystems and, above all, for the subsistence of many crops, due to their ability to pollinate flowers. Currently, the honey bee populations are decreasing due to colony collapse disorder (CCD). The reasons for CCD are not fully known, and as a result, it is essential to obtain all possible information on the environmental conditions surrounding the beehives. On the other hand, it is important to carry out such information gathering as non-intrusively as possible to avoid modifying the bees’ work conditions and to obtain more reliable data. We designed a wireless-sensor networks meet these requirements. We designed a remote monitoring system (called WBee) based on a hierarchical three-level model formed by the wireless node, a local data server, and a cloud data server. WBee is a low-cost, fully scalable, easily deployable system with regard to the number and types of sensors and the number of hives and their geographical distribution. WBee saves the data in each of the levels if there are failures in communication. In addition, the nodes include a backup battery, which allows for further data acquisition and storage in the event of a power outage. Unlike other systems that monitor a single point of a hive, the system we present monitors and stores the temperature and relative humidity of the beehive in three different spots. Additionally, the hive is continuously weighed on a weighing scale. Real-time weight measurement is an innovation in wireless beehive—monitoring systems. We designed an adaptation board to facilitate the connection of the sensors to the node. Through the Internet, researchers and beekeepers can access the cloud data server to find out the condition of their hives in real time.

## 1. Introduction

Insect pollinators are essential for terrestrial ecosystems and for many agricultural and farming businesses. They ensure the maintenance of certain environmental processes, such as plant reproduction. Bees are the most specialized insect pollinators due to their ability to transport and store pollen efficiently [1]. Thus, honeybees are not only important for their honey production but also for environmental balance, because they are essential to the pollination of the flowers of many crops [2].

In recent years, honeybee populations have experienced significant losses due to colony collapse disorder (CCD). The reasons for the CCD are still being debated. As a result, it is essential to obtain information to look for solutions to this syndrome [2,3]. Thus, bee colony monitoring—registering the largest possible amount of data but preventing the effects of handling on beehives—is important. In this way, it will be possible to obtain highly reliable information. Remote monitoring systems meet the necessary requirements to turn into a significant tool for the monitoring of bee colonies.

The use of precision apiculture allows us to monitor the beehives for many possible reasons, such as research, information about the daily management of bees by beekeepers, and learning how to reduce the resources and time assigned to tasks without reducing production.

Beehive monitoring allows us to monitor different parameters, such as the temperature and humidity levels inside the beehives, as well as the weight, sounds, and gases produced, which can generate important information. For example, these data can inform us on whether the beehives are swarming based on the temperature, whether any action is required from the beekeeper, whether the bees are affected by any disease, or even whether the hives are moving. This last application is very useful in areas where beehives can be stolen [4,5,6,7,8].

Different technologies can be applied to monitor the hives [9]. Reducing the cost and size of the sensors allows the possibility of deploying them in the countryside to extract information and collect data more easily. Initially, remote electronic monitoring of beehives had a mainly scientific application, allowing the monitoring of factors inside the hives, such as the temperature and the humidity, with minimally invasive methods. Nowadays, these innovations have begun to be adapted by beekeepers through practical applications that can provide remote information for the decision-making processes without having to inspect the beehives.

However, the monitoring of biological processes is not a trivial task, due to the fact that the behavior of the biological system and its reaction to human interaction is not predictable. The acquisition of data in industrial processes is a very common issue, and there are many commercial systems for such tasks. However, in the scope of biological processes, data acquisition becomes a complex task, especially in the case of honeybee hives, where any foreign object can turn into a threat to be neutralized by the bees.

In recent years, monitoring systems have greatly progressed due to the use of wireless-sensor networks (WSNs). A WSN is made up of embedded devices that can acquire data from different sensors, process them, and communicate with a computer with a cloud database. These devices are known as nodes or motes and are the main core of the Internet of Things (IoT). Humans have used WSNs in many daily life activities, such as agriculture [10]. They have been also used in health care, intelligent home monitoring, archeological monitoring system, and military and industrial applications [11,12,13].

WSNs are also beginning to be used to monitor honeybee colonies. The nodes of a WSN can be used to obtain data from the sensors monitoring the environmental conditions of a beehive (temperature, humidity, CO_2_, etc.) and even its weight [14]. The nodes connect and communicate through a gateway that can send the data from the motes to a base for storage and processing. This has increased the features of the monitoring systems, leading to what is currently known as precision apiculture. WSNs are a well-known alternative for distributed and remote sensing. These kinds of networks offer strong potential for beekeepers, due to the fact that they meet several significant requirements: (i) they are a minimally invasive method due to the small size of the nodes and sensors; (ii) they can be operational in remote areas for a long period of time due to their low energy consumption; and (iii) they allow real-time monitoring.

This work presents a wireless monitoring system for honeybee hives. The system architecture allows easy deployment in the field and ensures easy scalability. We used commercial nodes but adapted them to obtain measurements from the hives. We also developed an adaptor board.

The paper is organized as follows: Section 2 provides a short review of honeybee monitoring. Section 3 presents an overview on the architecture of the system. Section 4 describes the hardware and software implementation of the wireless node. Section 5 discusses power consumption and autonomy of the node. Section 6 describes the installation of the system in the apiary. Finally, Section 7 provides our conclusions.

## 2. Related Works

The interest regarding continuous honeybee colonies monitoring began during the first years of the *XX*-th century. In 1914, Gates published data on the temperature of a beehive manually collected every hour for several days in 1907 [15]. In 1926, Dunham measured the temperature inside a beehive by means of thermocouples [16]. The technological development of the sensors and electronic data-acquisition systems has allowed the improvement of measurement processes. In this way, different types of monitoring methods have been used, from simple observation of the data in the hive [17] to systems that are able to analyze those data [18]. Meikle and Holst summarized some of the continuous monitoring methods in [9], stating for each method the author, the measured parameters (temperature, O_2_, CO_2_, relative humidity, weight, vibration, etc.), and each method’s length.

One of the first electronic systems to monitor bees was Apidictor [19]. This system consisted of a low-pass filter for frequencies to detect the changes of sound that took place inside the hive up to two to three weeks before swarming.

In [20], Ferrari et al. analyzed sound recorded with a sound card in a computer and monitored the temperature and relative humidity by means of a datalogger. The sound was recorded through three omnidirectional microphones. In [21], Chen et al. described an imaging system located at the entrance to the hive to find out the number of times a bee entered and exited the hive. They used a tag attached to the bee’s body to identify the bee under study. In [22], Zacepins and Stalidzans monitored the wintering process of bees, measuring the temperature of the beehive. The data were sent to a computer for processing and to determine the status of the hive by means of the application of an algorithm. In [23], Heidinger et al. used a radio-frequency identification (RFID) system to find out the frequency and length of the nuptial flights of honeybee queens.

Meikle et al. used electronics balances connected to a 12-bit resolution datalogger to assess the evolution of the weight of a beehive. The system was powered by a solar panel. They recorded the weight of two hives hourly [24].

The improvement of the performance and size of the microcontrollers has allowed the development of low-cost beehive-monitoring systems based on Arduino^®^, Make^®^, Sparkfun^®^, and Adafruit^®^ [25]. An example of an Arduino-based monitoring system is that proposed by Sánchez et al. [26]. The system stored the temperature and relative humidity data in a microSD memory card by means of an Excel database. The beekeeper needed to go to the beehive colony and download the content of the SD memory card for each hive to his/her laptop to be able to process those data later on.

In recent years, honeybee hive monitoring systems based on WSNs have been used. These systems have the advantage of being able to carry out a non-intrusive remote monitoring of the hive’s environmental conditions. In [27], Kviesis et al. developed a system with a main unit that communicated wirelessly with each node located in the hive, concurrently acting as an Internet gateway. The system monitored the temperature and relative humidity using an integrated SHT15 sensor. The collected data were sent by the main unit to a remote database server. In [28], Zacepins et al. described a temperature-monitoring system based on Raspberry Pi acting as an Internet gateway. The temperature sensors located in the hives connected to the Raspberry Pi via a one-wire network. The authors highlighted the low cost of the solution if just one Raspberry Pi were used for all beehives.

In [29], Murphy et al. used a WSN to monitor a hive colony and collect the most important information on the activity/environment within a beehive, as well as its surrounding area. Each beehive was monitored by means of two nodes, and each of the nodes was implemented through a Waspmote^®^ by Libelium (Zaragoza, Spain). To monitor the honeybees in the hive, several heterogeneous sensors were used (temperature, CO_2_, pollutants, NO_2_, etc.). Due to the high number of sensors, two commercial expansion boards were used, one for each Waspmote. Each sensor was read at a frequency of six samples/day. The collected samples were stored on an SD card for backup and were broadcasted over a Zigbee network once every 24 h to the base station. The base station acted as a bridge, sending the data to the remote server over a 3G/GSM connection. This monitoring system was used by Murphy et al. to propose several algorithms to automatically detect changes in the hive and warn the beekeeper of potential colony threats [30]. Based on the CO_2_ levels of the hive, an algorithm was proposed to predict weather conditions.

Kridi et al. developed a system based on the Arduino platform to measure the temperature of the beehive by means of the LM35 sensor [31]. The data were sent wirelessly through an XBee-Pro module to a base station desktop PC connected to the Internet. The collected data were processed to find patterns that determined the thermal stress in the hive to detect potential absconding conditions.

Joe-Air et al. developed a system to monitor the frequency of bee activity to relate the activity to environmental factors [32]. It was a wireless system that monitored the frequency of bee entrances and exits from the hive together with the temperature and humidity inside and outside the hive. According to the authors, the accuracy obtained from the frequency depended on the activity of the hive, with an average error of 10% and a maximum value of 20%.

## 3. System Architecture

Beehive-monitoring systems must meet several significant requirements: (i) they must use a minimally invasive method; (ii) they must be operational in remote areas for long periods of time, and (iii) they must allow real-time monitoring. In [33], Kviesis and Zacepins described different automatic monitoring–system architectures for real-time beehive monitoring, distinguishing their advantages and disadvantages. In our case, we have chosen a three-level hierarchical model based on wireless communication. It is an easily adaptable model for any geographic distribution of hives, is scalable at any of the three levels, and meets the requirements indicated above.

**Figure 1:**
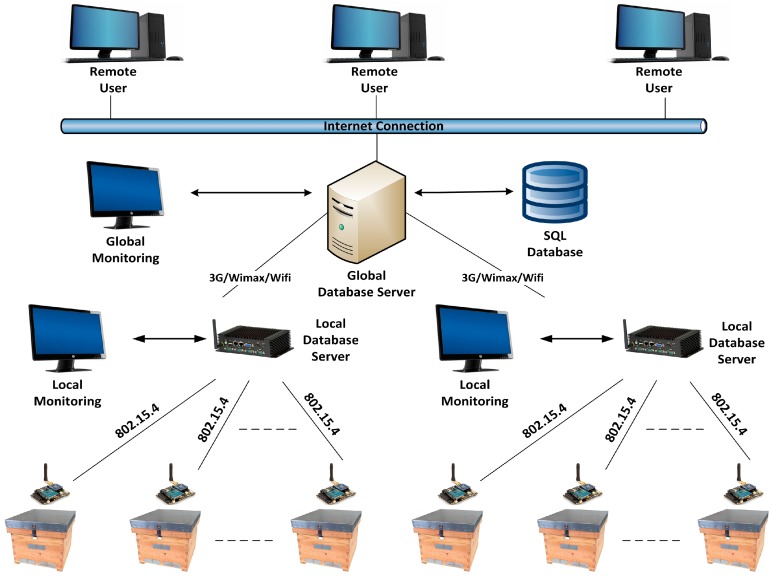
Architecture of the WBee system.

Figure 1 shows the general structure of the beehive-monitoring system, WBee, described in this work. At the lowest level, we can find the wireless nodes that monitor the hives and send the data in wirelessly using IEEE 802.15.4. At the intermediate level, managing the wireless networks of a group of beehives, we can find an industrial computer with 802.15.4 communication that also acts as the network’s coordinator. This computer contains an application that collects the information sent by the nodes of each beehive and stores them in a local database. The communication with the global-server database is executed through another communication network that allows for greater distances, such as 3G/GPRS, WIFI, or WIMAX. Lastly, the upper level contains the cloud-server database, which groups several beehives and contains a copy of the local database of each group of hives.

The local computer for each apiary can work in isolation, since it can work as a database server. This characteristic ensures easy deployment and scalability of the system. Thus, new apiaries can be debugged before being integrated into the system. This local computer at the intermediate level is an important difference from other wireless monitoring systems in which a sink mote does not store information and is only used as a gateway between the WSN and global database server. Later, a more detailed description of each part will be provided.

**Figure 2:**
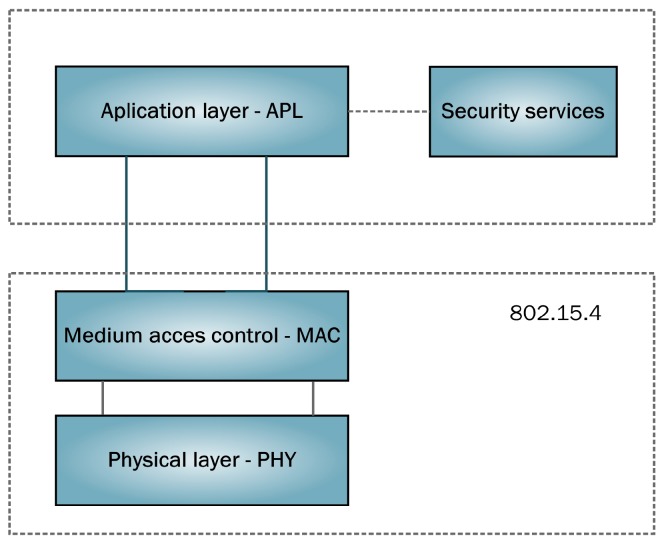
Layer model.

The 802.15.4 standard is framed within the area of the wireless personal area networks (WPANs), with low consumption and cost. As a result, it has increasingly gained relevance in the industrial domain. Standard 802.15.4 only defines the physical and media-access control layers. Our applications directly communicate with the MAC layer, as shown in Figure 2.

Standard 802.15.4 defines two types of topologies: peer-to-peer and star networks. The peer-to-peer topology allows each node of the network to communicate with any other node, provided that the node is within its range. The star topology allows the establishment of communication among the devices and a sole central node that acts as the network coordinator. The network coordinator is responsible for starting and finishing the connections.

For this apiary monitoring system, we chose a star topology. Thus, the local computer acts as the coordinator. All nodes installed in the beehives must be accessible for the coordinator. This restriction does not imply a problem, since the beehives are on field and as a result, there are no significant obstacles usually. The use of this topology simplifies the communications, as we do not need nodes acting as routers or a network layer for frame routing.

Each node has two unique identifiers—the radio module’s MAC address and a node identifier (nodeID)—that allow it to be located by the application, similar to a hive address. In this way, when a radio module is replaced, only the same nodeID needs to be selected in the node. The nodeID has a length of 16 bits and can select up to 65,536 beehives. The application in the local computer collecting the data sent by each hive only needs to know the nodeID. This application maintains a table with the MAC addresses and the nodeID. These tables are dynamically created when the network is initialized, when a node is connected, and so on.

### 3.1. Wireless Node

The wireless node, which will be described in detail in Section 4, is based on the Waspmote by Libelium [34], but with an additional adapting board especially designed for the connection of three humidity and temperature sensors and the interconnection of a scale.

**Figure 3:**
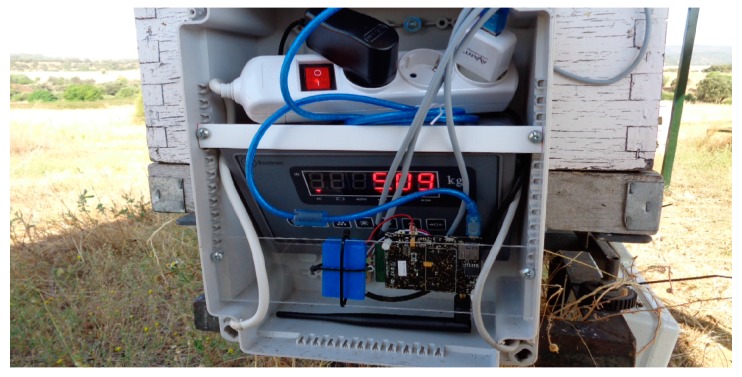
Photograph of one of the nodes installed in the beehive.

The system is designed so that each beehive has its own wireless node and scale. Figure 3 shows a photograph of the system attached to the beehive. The system is protected with an IP65 box. The weighing scale display has been kept so that the beekeeper can check the weight of the hive onsite.

### 3.2. Local Data Server, and Supervisory Control and Data Acquisition System

**Figure 4:**
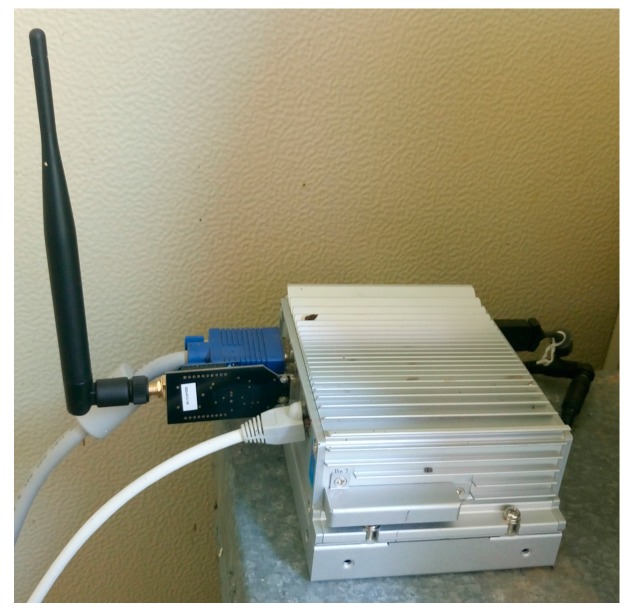
Local database server.

Two applications are executed on the local computer located in each apiary: a supervisory control and data acquisition system (SCADA), which synchronizes, requests, and processes the data of the node in each beehive, and MySQL, the world’s most popular open source database [35]. MySQL is also executed in the cloud data server, and a replica of the local database of each hive is carried out. This server guarantees an extra level of security in the event of a communications failure, since it stores and sends the data acquired in each beehive to the cloud. The local computer is an embedded industrial computer, which shows a higher protection against environmental conditions, as it must be placed beside the beehives. The computer communicates directly with the coordinator node of the wireless network using a USB interface, as shown in Figure 4. We used Libelium’s “XBee USB-Serial gateway” module as the coordinator of the network and gateway [36].

**Figure 5:**
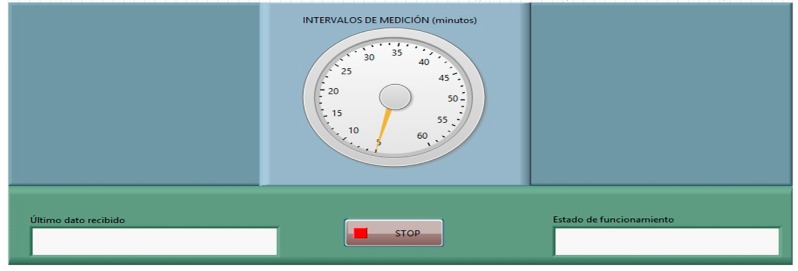
SCADA user interface.

The software used for the execution of the SCADA was developed under Laboratory Virtual Instrumentation Engineering Workbench (LabVIEW), an environment developed by National Instruments (Austin, TX, USA) that uses a graphic programming language. The SCADA communicates with MySQL using the LabVIEW SQL/ODBC library to store the collected information [37]. The application has one simple user interface in which it is possible to control the data acquisition interval for the data in the nodes (Figure 5), and a user-friendly part is responsible for the communication with each of the nodes, the data processing, and their storage.

**Figure 6:**
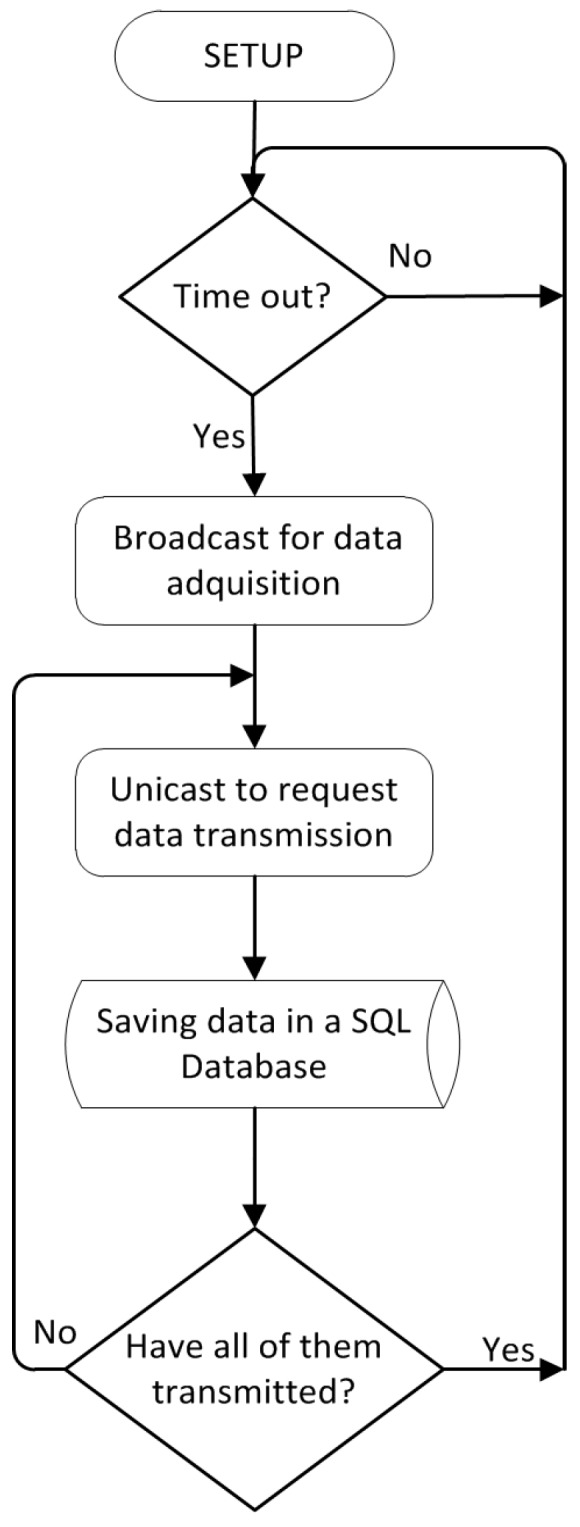
SCADA flowchart.

The operation of the SCADA application is shown in Figure 6. Firstly, certain parameters are set in the application, such as the initialization of the coordinator node. Then, the application waits for the measurement interval programmed by the user. Once the wait is over, the broadcast for the beginning of the data acquisition is sent to the nodes so that all acquired samples are correlated, and afterward, the samples are requested from each node. If no data have been received from a specific node, they are requested again up to three times.

### 3.3. Cloud Database Server

**Figure 7:**
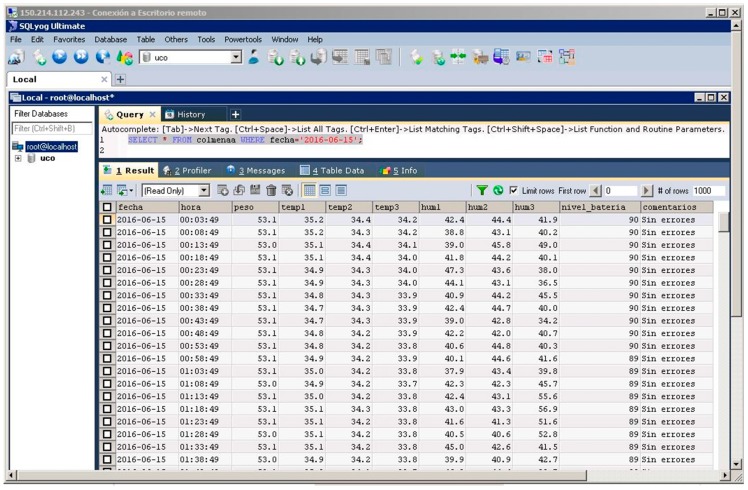
View of a query to the cloud database server.

A replica of the local database of each beehive is carried out in the cloud database server. This server ensures an extra level of security, providing both a backup and access the information of the beehives and sending the collected data to the cloud. As a result, through this server, the beekeeper can access all data about the beehives from anywhere through the Internet and can also receive alarms or other actions required by the beehives with an automated analysis of these data. Figure 7 shows the temperature and humidity data of a beehive stored in the cloud database server. The battery level and information regarding whether there are errors in the data acquisition from node are also stored in this database.

## 4. Implementation of the Wireless Node

The wireless node replaces the data acquisition and processing designed to monitor the thermoregulatory capacity of the honeybee colonies in hives with open-screened bottom boards [26]. A new node and a different system architecture have been designed. In the previous system, three LM35 temperature sensors were installed in different areas of each beehive together with two SHT15 sensors, which can measure the hive’s temperature and humidity. An SHT15 was placed on the upper side of the beehive by the hole leading to the air chamber located under the lid. The other SHT15 sensor was placed under the hive to measure the outer temperature and humidity, allowing it to compare these data with the temperature and humidity inside the beehive. This system was installed in 10 hives. Five of the hives had conventional closed bottom boards and the other five had open-screened bottom boards. The electronic system was based on the Arduino platform and stored the data in a microSD memory. The beekeeper had to manually copy the content of the microSD card into his/her laptop for it to be processed later on. Thus, a new system based on a wireless sensor network was designed so that the measurement data obtained by each node were transmitted to a local server and from there, to the cloud database server. In this way, the beekeeper can see and download the measurement data for temperature, relative humidity, and weight from the Internet and more importantly, do so non-intrusively, not interfering with the work conditions of the beehive.

The wireless node is based on the Waspmote mote by Libelium [34]. We selected this mote because it has the necessary characteristics to implement the most suitable wireless node to monitor a beehive. Among these characteristics, the following are worth mentioning: ultra-low power (7 µA hibernation mode), allowing the connection of any sensor using any wireless technology to any cloud platform; the ability to program it on the air (OTA); encryption libraries (AES, RSA); and several peripherals that will be mentioned below. Another advantage is that it allows the connection of several radio modules, depending on the transmission distance. In this way, by connecting the most appropriate radio module, it is possible to use long-range technologies (3G/GPRS/LoRaWAN/LoRa/Sigfox/868/900MHz), and in the case of isolated beehives, medium-range technologies (ZigBee/802.15.4/Wi-Fi) and short-range technologies (RFID/NFC/Bluetooth 4.0). On the other hand, the Waspmote platform includes the Waspmote-IDE (integrated development environment) used to program Waspmote [38]. This IDE offers a series of function libraries to easily control the different peripherals, such as the microSD card, RTC, UART, and the digital I/O.

Murphy et al. [29] also used the Waspmote for the implementation of a heterogeneous WSN, which monitors the internal conditions of a beehive colony though a diverse set of sensors. Murphy et al. used the Plug & Sense! version of Waspmote and the sensors offered by Libelium for that version with the appropriate connectors. This version of Waspmote has a drawback: If many sensors of the same type and a weighing scale need to be connected through a serial interface RS-232, as in our case, it is necessary to use two Waspmotes per node, as done by Murphy et al. in her implementation. Thus, we have decided to use the standard version of Waspmote and develop an adapter board in order to connect all sensors through the digital I/O ports. This board also includes the adaptation circuit for levels RS-232 to 3.3V CMOS to connect the weighing scale to the Waspmote UART. The resulting wireless node will be called UcoBee. We will describe both the hardware and the software of the UcoBee wireless node below.

### 4.1. Hardware Description Node

**Figure 8:**
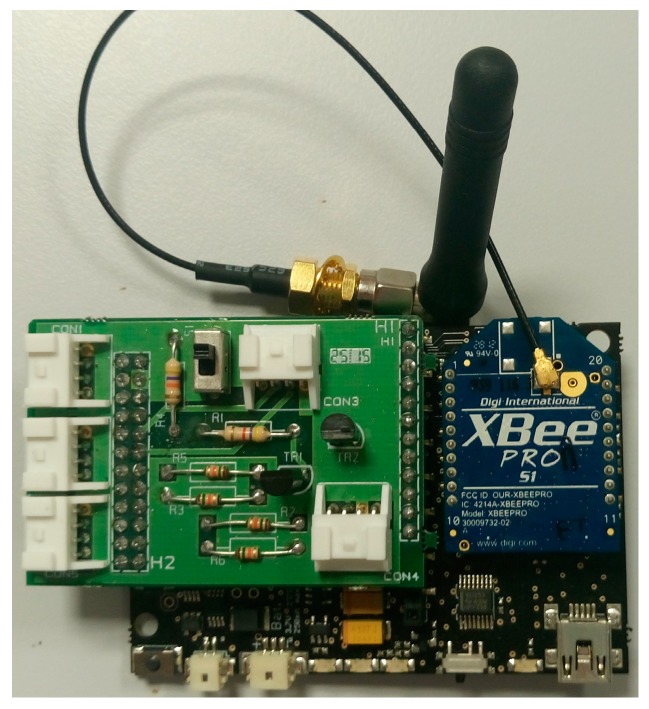
UcoBee Version 1 wireless node.

This section will include a description of the hardware of the UcoBee wireless node, Version 1, shown in Figure 8.

**Figure 9:**
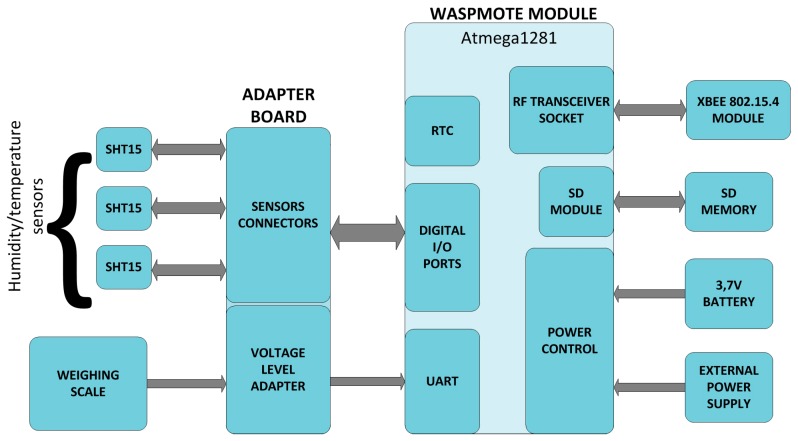
UcoBee block diagram.

The block diagram of the wireless node is shown in Figure 9. The following blocks can be distinguished: Waspmote module, XBEE PRO module, adapter board, microSD memory card, battery, and external power supply.

#### 4.1.1. Power Supply and Battery Block

Due to the closeness of the main 230 VAC power supply to the beehive apiary, two power supply units, which will be mentioned below, were used. If this were not available, the node could receive its power supply through a system based on a solar panel.

For the operation of the system installed in each beehive, two power voltages were needed: on the one hand, a power supply of 5 VDC/1 A with a miniUSB connector to supply the power to the Waspmote and another one of 12 VDC/0.5 A for the weighing scale with a female jack connector. The weighing scale includes a rechargeable lithium-ion 6 V and 2500 mAh battery, and a 3.7 V/2300 mAh rechargeable lithium-ion battery was connected to the Waspmote. Both the Waspmote and the weighing scale included a control circuit for loading the battery with the power supply. In this way, the wireless node could continue operating even if there were a power outage. If this happened, the measurement data would be stored on the microSD card and later on, when the power returned, they would be transferred to the local computer. The addition of the batteries, together with the power supply through a solar panel, allow the set of the wireless node and the weighing scale to be used in areas where there is no available electricity network near the apiary.

#### 4.1.2. Humidity and Temperature Sensors

In order to measure the humidity inside the beehives, three SHT15 sensors by Sensirion were used [39]. They were chosen due to their excellent reliability and stability and their low power consumption. These sensors allow the measurement of temperature by means of a band-gap sensor and of the relative humidity through a capacitive sensor. The SHT15 sensor includes a 14-bit analog/digital converter (ADC) and a serial interface circuit. The ADC converts the signal generated by both sensors into a digital signal and transfers the results of the conversion through the serial interface. In this way, a better-quality signal, quick response, and better immunity to external disturbances (EMC) are obtained. Within the SHT1x family, the SHT15 is the sensor with a higher accuracy, providing an error of ±0.3 °C for temperature and ±2% for relative humidity.

With the Plug & Sense! Version of Waspmote by Libelium, we would have needed to use the 808H5V5 humidity sensor and the MCP9700A temperature sensor. These sensors are analog, so they would have needed to be connected to the analog channels of the ATmega1281. In order to obtain the highest possible resolution in the ADC of the microcontroller, Waspmote Plug & Sense! must include a signal amplification board. Regardless, due to the fact that the ADC of the ATmega1281 is a 10-bit converter, a worse resolution would be obtained; since SHT15 sensors include an internal 14-bit ADC and because they are beside the measurement sensor, a better signal-noise ratio is obtained. On the other hand, the cost of the node is reduced, as Waspmote Plug & Sense! is more expensive.

**Figure 10:**
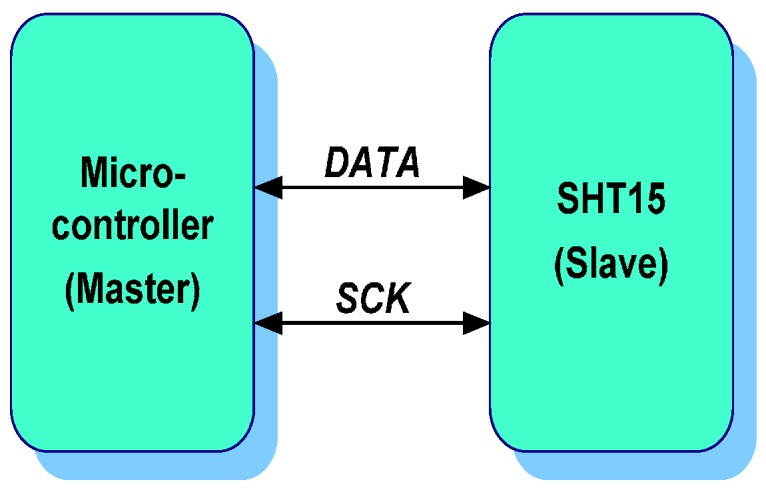
Communication interface between Waspmote and SHT15.

The reading of and request for data is executed through a serial interface based on I²C known as Sensibus, as shown in Figure 10. This interface uses two signals: SCK and DATA. SCK is used to synchronize the communication between the microcontroller and the SHT15 sensor. The DATA pin is used to transfer data to or from the sensor. The SHT15 generates the measurement data with the falling edge of SCK. Thus, the microcontroller needs to read the data bits in the rising edge of SCK. The implementation of the communication protocol has been implemented through software using two I/O pins of the microcontroller.

The output digital signal is internally calibrated by means of calibration coefficients programmed in an internal OTP memory in the chip. This increases the stability of the signal to the internal variations due to the changes in temperature.

**Figure 11:**
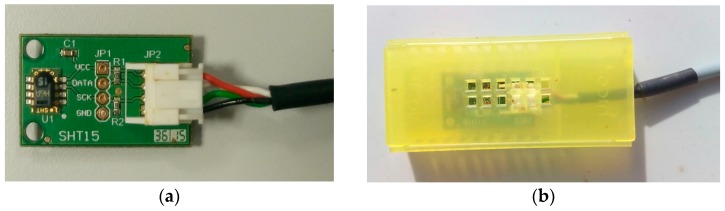
(**a**) SHT15 sensor mounted in PCB; (**b**) Cage for the protection of SHT15 sensors.

To reduce cost and obtain a reliable and easy connection for the sensor, we designed a PCB in which the SHT15 has been mounted and a male connector with a block device to connect it to the expansion board has been included, as can be seen in Figure 11a. Due to the tendency of bees to cover any foreign object inside their hive with propolis (resinous material collected by bees from bugs from trees and used as a cement to repair and maintain the hive), the sensors have been protected by enclosing them in perforated queen expedition cages Nicot^®^, as shown in Figure 11b.

#### 4.1.3. Weighing Scale

The weighing scale consists of a metallic 50 cm × 40 cm frame with a 150 kg load cell associated. This weight is more than enough, as the weight of a beehive can be up to 80 kg. The load cell is connected to a BR80 display by Baxtran [40]. The display has a six-digit screen, so it can display up to 100,000 different values. The resolution can be set up from 1 kg to 5 g. In our case, we have selected a resolution of 100 g, as we considered it to be sufficient for later analysis of the measurements, so that it is possible to determine, for example, whether the blooming period (and as a result, the bees’ honey production) is over.

**Figure 12:**
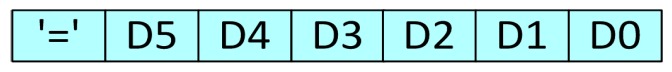
Frame format sent by the weighing scale.

The BR80 display has a DB9 connector that periodically sends the weight values through a series RS-232 interface, according to Figure 12. The frame consists of seven bytes. The first character sent is “=”, which is used to synchronize with the receiver (Waspmote) and to note that the six digits regarding the value of the weight will be then sent. The first digit is most significant, and as specified by standard RS-232, all of the characters are codified in ASCII code. The transmission speed can be configured from 1200 to 9600 bauds. The lowest speed has been selected in our case to allow the Waspmote to receive the measurements from the three SHT15 sensors without overflow occurring in the reception buffer of its UART.

#### 4.1.4. Waspmote Module

The most important component of the wireless node is the Waspmote mote by Libelium. This mote is based on the ATmega1281 microcontroller by Atmel (San Jose, CA, USA). The ATmega1281 includes a 128 kB FLASH EPROM for the memory of the program, 8 kB SRAM to store data, a module to mount a microSD card, and a Real Time Clock (RTC). The size of the Flash memory is more than enough to store the application that controls the node, just as happens with the SRAM to temporarily store the data obtained by the sensors. In case more storage capacity is necessary, the microSD card can be used.

The Waspmote has a built-in RTC. The RTC we chose was the DS3231SN by Maxim (San Jose, CA, USA) which operates at a frequency of 32.768 Hz. This RTC can be programmed to generate an alarm to collect the values of the sensors and execute other actions, such as sending the values of the measurement data to the local server. Also, it allows Waspmote to use energy-saving modes and allows it to wake up at the required moment.

Another advantage of the Waspmote is that it has several expansion connectors. The adapter board, which will be described in a later section, will be connected to these expansion connectors. The Waspmote allows three power supply options: battery (3.3–4.2 V), USB connector (5 V), and solar panel (6–12 V @ 280 mA). When in operation, its consumption is 17 mA, whereas in the sleep mode, it is 33 µA, although due to the closeness of the mains to the colony, the power consumption does not represent a problem. Otherwise, the battery and the solar panel to charge the battery could be used, as was mentioned above.

An XBee-802.15.4-Pro module [41] was installed on the radio socket, with a maximum power consumption during transmission of 100 mW and a transmission frequency of 2.4 GHz. If a 5 dBi antenna is connected, its range reaches 7 km, allowing for perfect communication with the local server, as this is usually at a distance of no more than 20 m. If the bee colony had to be placed in a rural area far from the local server, a long-range radio module, such as GSM/GPRS, LoRaWAN, or LoRa, could be used.

Waspmote has a built-in LIS3331LDHS acceleration sensor by STMicroelectronics (Geneva, Switzerland), which informs the mote of acceleration variations experienced on each one of the three axes (X, Y, Z). As a result, it is possible to know whether anyone has moved the beehive or even worse, if anyone has tried to steal it. On the other hand, it is possible to connect an optional module with a GPS receiver to a Waspmote, which allows the beekeeper to know the exact outer location of the mote at any time. These two options were not used in the current application, but we are planning to include them in a later version to find out whether the beehive has been stolen and to know its exact location when searching for it. The accelerometer and the GPS module also affected the final choice of the Waspmote for the implementation of the wireless node.

#### 4.1.5. Adapter Board

This board has been designed to be able to connect the three SHT15 temperature and humidity sensors and the RS-232 transmission serial signal generated by the weighing scale. Four connectors 4-way of 2 mm pitch for PCB assembly in right angle are used. These ensure a safe connection of the cable to the board, as they have a locking mechanism. The cable used for the connection is a twisted pair cable with sheet jacket by Alpha Wires (Elizabeth, NJ, USA), and the conductors are stranded wire tinned copper 24 AWG cables.

As mentioned, another function carried out by the adapter board is to adapt the RS-232 levels of the weighing scale’s transmission signal to the UART of the Waspmote, which is 3.3 V CMOS. The weighing scale does not include a RS-232 compatible driver, but it generates the signal with an operational amplifier supplied with a unipolar supply voltage of 10 V. Thus, if the transmission signal is idle or represents a data bit with value 1, it has a value of 0 V, but if it begins to transmit the start bit, or if it represents a data bit with value 0, it has a value of 8 V. As a result, the transmission signal meets the RS-232 standard for value 0, which corresponds to a voltage higher than +3 V. The fact that value 1 is represented by means of a voltage of 0 V does not imply any problem in our design, even though it does not meet standard RS-232, which states that the voltage must be lower than −3 V. On the contrary, as will be explained below, it will allow us to simplify the adapter circuit.

**Figure 13:**
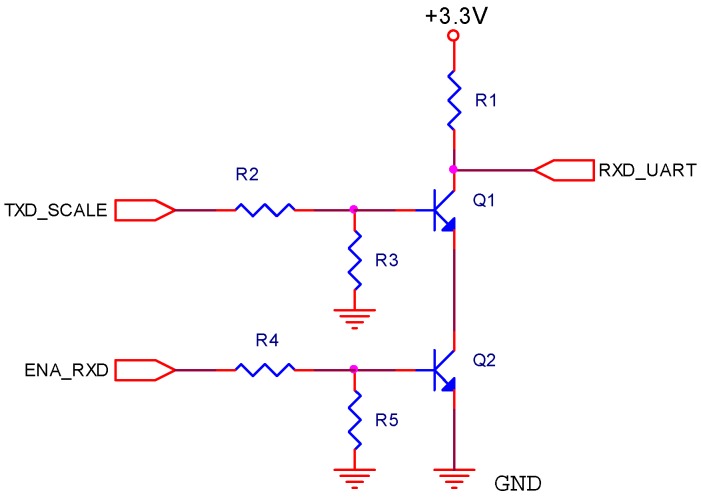
Adaptation circuit of levels RS-232 to 3.3 V CMOS.

As Waspmote uses a supply voltage of 3.3 V, the reception input voltage range of the UART varies from 0 V to 3.3V. Therefore, the transmission signal of the weighing scale (8 V) cannot be directly connected to the UART (3.3 V max.). To adapt the voltage levels, the circuit shown in Figure 13 was designed. It has two bipolar transistors which, besides reducing the voltage from 8 V to 3.3 V approximately, inverts the logic value to meet the RS-232 standard.

To simplify the analysis, let us suppose that the Q2 transistor is saturated. Then, its function will be exposed and its operation will be analyzed. If the TXD_SCALE signal is at 0 V (idle or data bit with value 0), it does not drive transistor Q1. Thus, RXD_UART will be at a high logic level (3.3 V) through resistance R1. If, to the contrary, TXD_SCALE is at 8 V (start bit or data bit with value 1), the transistor gets saturated, and RXD_UART will be at a low logic level.

As the weighing scale periodically transmits the value of the weight every second and our application takes weight into consideration every 5 min, another transistor in a totem-pole mode was connected with the previous one. The base is controlled through a digital output of the Waspmote, which acts as reception enable. If ENA_RXD is at a low logic level, the Q2 transistor will be cut off. Then, independently from the state of the Q1 transistor, RXD_UART will be at a high logic level through R1 resistance. If on the other hand, ENA_RXD is active at a high logic level, the Q2 transistor gets saturated and the value of RXD_UART will depend on TXD_SCALE, as previously mentioned. In this way, the power consumption is reduced, and since Q2 is cut off when the Waspmote is not reading the weight data, there will be a high resistance between GND and the positive pole of the supply voltage, despite the saturation of Q1.

As previously mentioned, the Waspmote includes a microSD card. This is used to store the data regarding the measurements of the sensors and the weighing scale if communication with the local server is lost. In the expansion board, a switch, connected to a digital input of the Waspmote, has been included to enable the writing operation and to inhibit it, in order to remove it safely.

Additionally, a connector of the same type as those used to connect the sensors and the weighing scale has been added to the adaptor board. This connector is connected to the Waspmote microcontroller’s two analog channels. In this way, it will be possible to connect two analog output sensors (e.g., the CO_2_ sensors or a microphone to record sound). This connector can be power supplied if a signal amplification of the sensors should be necessary.

### 4.2. Software Node

The application executed in the wireless node was developed using the IDE-Waspmote open source-based environment. C language is used and several libraries are provided by the manufacturer. As in all Arduino environments, the application has a SETUP and a loop, which is indefinitely executed.

Once the node has been initialized and associated with the wireless network, the application is in standby, waiting for the reception of wireless orders. If no communication is received, the node collects samples using either the last programmed period or a default one and stores the samples on the microSD card. If there are no communication problems, the node will wait for the order to collect samples. In this way, we managed to get all network nodes to collect the samples at the same time. Then, the node waited for its samples to be requested. The node always works in this way as a slave of the computer in a master/slave model. The node has an RTC, which is synchronized with each reception-request packet. In each packet sent by the node with the collected data, the timestamp of its acquisition is included.

The RTC is necessary for the node to continue the data acquisition—even though there is no communication, as mentioned above—as together with the data, the time when the samples are acquired is also saved in the microSD card.

**Figure 14:**
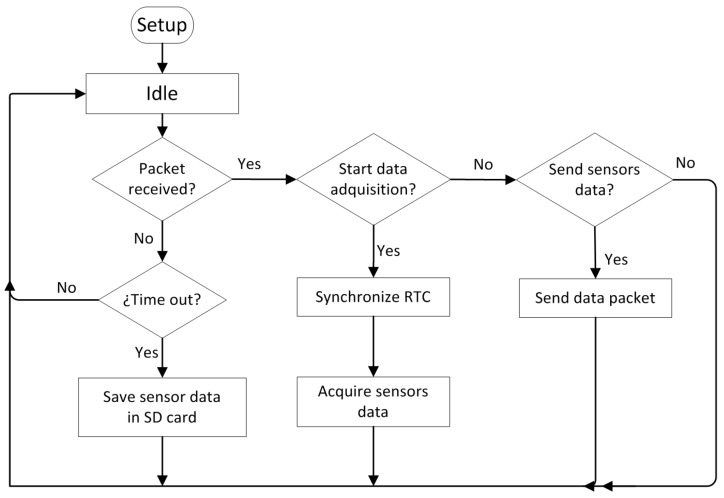
Waspmote program flowchart.

Figure 14 shows the flowchart of the application executed in the node. In normal operation mode, the node waits until it receives a broadcast sent by the local computer to collect the samples. Then, the node remains in standby, waiting to receive a new packet requesting the collected samples and responds with the samples and the timestamp.

## 5. Power Consumption and Autonomy of the Node

Each wireless node has its own backup battery. In normal conditions, the node is powered from another source of energy (solar panel or power grip). The Waspmote includes a control circuit for loading the battery with the power supply. This section describes the autonomy of the UcoBee node when the main power fails. A study of power consumption of the node will be carried out.

Table 1 shows the consumption indicated by the manufacturers. Since the consumption of SHT15 sensors is indicated for a 5 VDC supply, the consumption for 3.3 VDC is expected to be lower. The average consumption of the SHT15 indicated in the table is obtained by taking a measure per second.

The moment of acquisition and RTC of the nodes are synchronized with the local server, so the node can be kept in sleep mode and awakened before the acquisition request. This is possible using the RTC included in the Waspmote. Because physical magnitudes vary slowly over time, a 5-min minimum period of sampling has been considered, although the SCADA program that runs on the local server can be configured between 5 and 15 min. In order to reduce the power consumption of the node, once the measures have been acquired and sent to the local server, it goes into sleep mode for 4 min, so the node is in active mode for about a minute. Once the node is awakened, it performs the following operations: (1) Wait for the acquisition request, so the Xbee module is in reception mode; (2) Acquire the data from the SHT15 sensors; (3) Read the weight of the scale; and (4) Transmit the data to the local server, so the Xbee module is in transmission mode, and finally, the node goes into sleep mode.

It is necessary to distinguish two cases for the power consumption in active mode since there is a small difference in power consumption depending on whether or not the node has lost communication with the local server. The power consumption is higher when the Xbee Pro module is in transmission mode. If the communication is lost, the data is stored on the microSD card and the Xbee module does not transmit. In this situation, consumption is slightly lower.

Table 2 shows the experimental power consumption of the node. The measurements were performed on the network installed in the laboratory that was used to test the system. The network consisted of three nodes and a local database server. The measurements were performed with the active node acquiring data continuously and sending them to the local server. In the first two cases, the node did not transmit the acquired data, with the Xbee module remaining in receive mode. For the latter case, the nodes transmitted the data acquired continuously. The measurements were made observing the current consumption of the three nodes and calculating the average value. As we discussed in this paper this is not the average consumption in our application because the node is in sleep mode at least 80% of the time.

**Figure 15:**
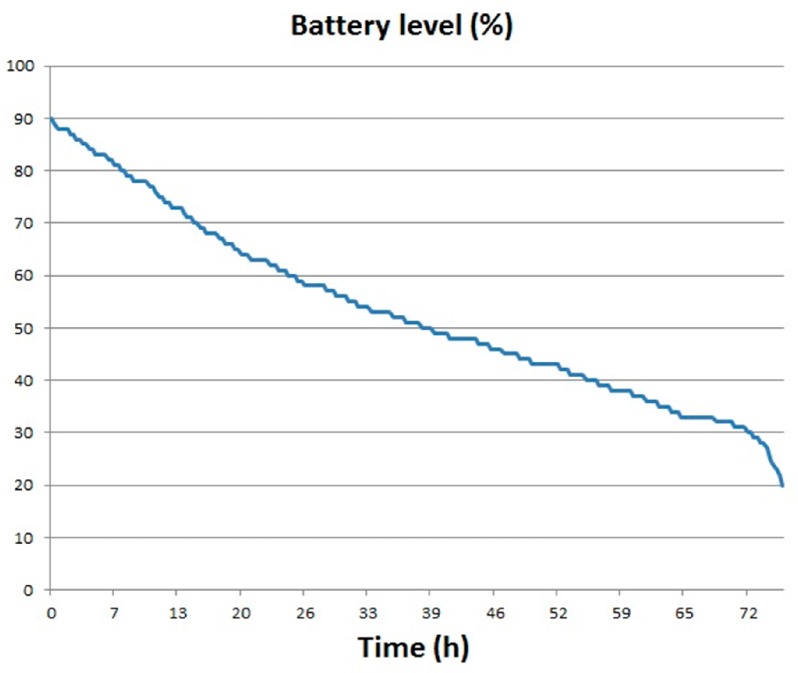
Battery level.

One of the most important aspects of the installation of the sensors network deployed in beehives is to know the battery autonomy when there is an outage in the power supply. To measure the autonomy of the nodes the main power to ten nodes installed in the apiary was removed for 75 h and the local database server was shut down. During that time, the sequence of operations is similar to the one described above, except that the Xbee module does not transmit data and the samples are stored on the microSD card. With a rechargeable lithium-ion battery of 3.7 V/2300 mAh, the battery level of the ten nodes that were analyzed fell an average of 70%, as shown in Figure 15. This time of autonomy is enough to solve the problem since the global server of the Wbee system generates an alarm when losing communication. Therefore it was not necessary to install a battery with higher capacity.

## 6. System Installation and Results

**Figure 16:**
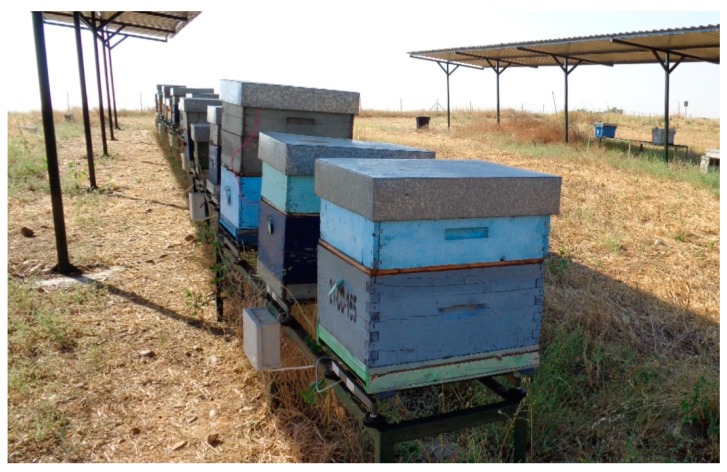
Photograph of the apiary.

This system is being used in 20 *Apis mellifera iberiensis* bee colonies located in an experimental apiary in the University of Córdoba (Córdoba. Spain; 37°55′33.5′′ N, 4°43′26.1′′ W). A node has been installed on each beehive. The colonies were formed during spring 2016. The bees were housed in Langstroth beehives placed on supports 50 cm over the floor, as shown in Figure 16.

**Figure 17:**
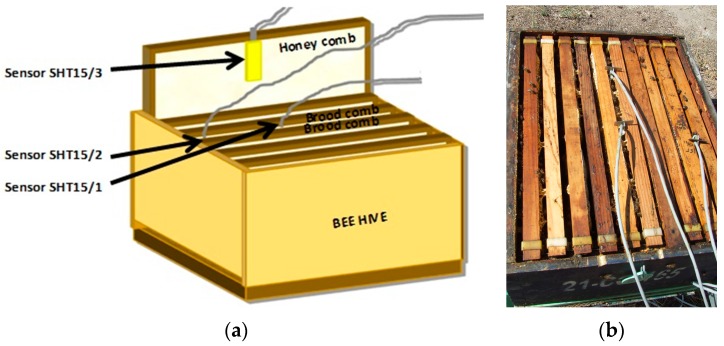
(**a**) Location of the SHT15 sensors in the hives; (**b**) Photograph of the hive.

Three SHT15 temperature and relative-humidity sensors were installed in each beehive in different locations: (i) middle of the brood area; (ii) area with honey/pollen reserves in the periphery of the same brood comb; and (iii) honeycombs separated from the brood combs. All of the sensors were located 12 cm below the top of the comb, as shown in Figure 17a,b. A node with just one SHT15, protected from environmental conditions, was installed to measure the temperature and humidity outside the hives. Each beehive was installed on a scale connected to the wireless node to weigh it in real time. The wireless node and the display of the weighing device were protected inside an IP65 box by each beehive. Three sensors were installed because according to studies carried out they show significant variations in relation to the thermal regulation that the bees carry out in different zones of the hive [26]. Thus, in the central brood area of the colony the temperatures are stable between 34–35 °C, which is the ideal temperature for the development of the bee larvae. However, as we move away from the brood area, temperatures are becoming less stable and therefore it is more similar to those outside the hive.

The local data server is correctly protected in a portable hut with an electric power supply. The cloud data server was installed in a building of the University Campus of Rabanales at the University of Cordoba.

Although the system began testing in September 2015, we chose to monitor the response of the bees to a sunflower bloom at the beginning of summer 2016, between 1 June and 2 July. The temperature, humidity, and weight in the hives were registered every 5 min.

WBee provided a large amount of very useful experimental data for researchers and beekeepers. As an example, three graphs of the average evolution of four beehives during the evaluation of the hives during the sunflower bloom are shown below.

**Figure 18:**
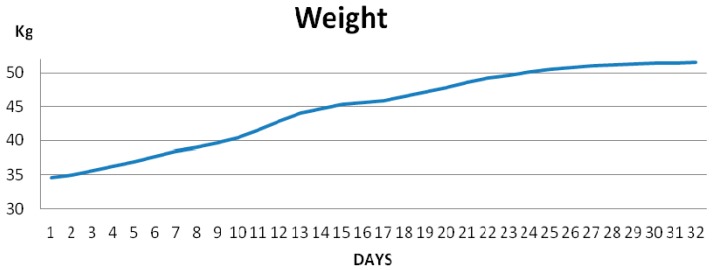
Average evolution of the weight (kg) of four beehives over 32 days.

Figure 18 shows the average evolution of the weight of four hives over 32 days. From Day 26 onward, it is possible to see that the weight of the beehives stabilizes, and this is interpreted as the end of the blooming period, implying that the beekeeper can now remove the honey harvest from the hives.

**Figure 19:**
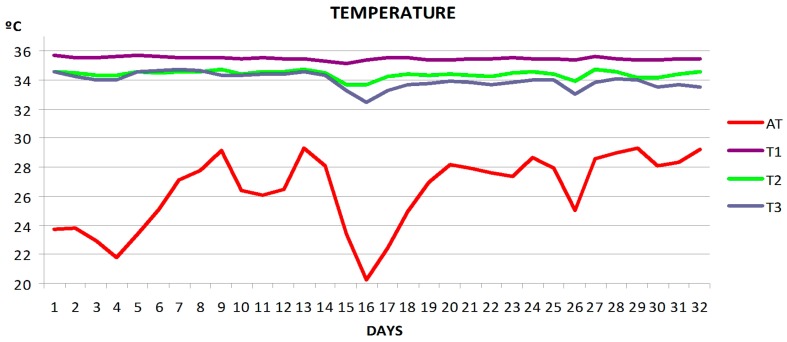
Average temperature per day of four hives over 32 days. Temperatures were registered at ambient (AT) and three areas inside the beehives: brood area (T1), area with honey/pollen reserves of in the periphery of the brood comb (T2), and in honeycombs (T3).

Figure 19 shows the temperature data registered during the experiment (32 days). The data show the ambient temperature outside the beehives (AT) and temperatures recorded in different areas of the beehives: (T1) middle of brood area; (T2) area with honey/pollen reserves in the periphery of the same brood comb; and (T3) honeycombs separated from the brood combs. The data correspond to the daily average temperature of four hives. Environmental temperature affected the temperatures inside the beehive, although it has been noted that the sensors placed in the middle of the brood area recorded higher and more stable mean temperatures within the hives.

**Figure 20:**
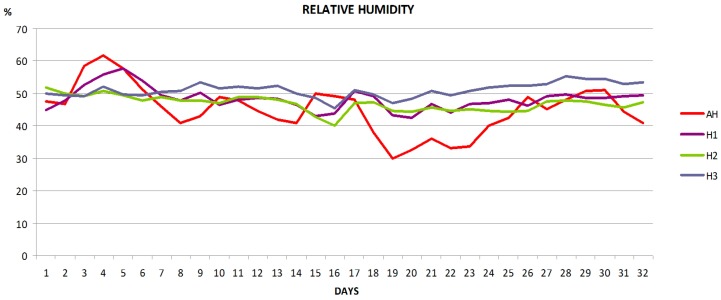
Average humidity per day of four hives over 32 days. Humidity was registered at ambient (AH) and three areas inside the beehives: brood area (H1), area with honey/pollen reserves of in the periphery of the brood comb (H2), and in honeycombs (H3).

Figure 20 shows the ambient humidity outside the beehives (AH) and humidity recorded in different areas of the beehives: (H1) middle of brood area; (H2) area with honey/pollen reserves in the periphery of the same brood comb; and (H3) honeycombs separated from the brood combs. The data correspond to the daily average humidity of four hives. The humidity inside of the beehive stays more stable than the outer humidity.

## 7. Conclusions

We have designed a low-cost, reliable beehive-monitoring system based on a WSN to measure the temperature, relative humidity, and weight of beehives in real time and non-intrusively. Unlike other beehive-monitoring systems, WBee performs the synchronized acquisition of samples from all hives of an apiary. This aspect is fundamental for future analysis of the data and their comparison between different hives. It will also allow researchers to compare physical magnitudes that change value quickly. Real-time weight measurement of the hive is an innovation in wireless beehive-monitoring systems.

WBee saves the data in each part of the network if there are failures in communication. In addition, the nodes include a backup battery for further acquisition and storage of data in the event of a power outage that could be sent once communication is reestablished. The use of a local database server is a novelty respect to other honeybee wireless monitoring systems. Other systems use a sink mote from the node manufacturer, which only works as gateway, and thus it does not maintain the database synchronized with the cloud database server. WBee obtains more information on the conditions of the hives than other systems because it monitors the temperature and relative humidity of the beehive in three different spots.

We managed to use just one Waspmote node per hive, as we designed an adapter board for the connection of the SHT15 sensors and RS232 interface of the weighing scale. The adapter board includes a simple adaptation circuit of RS232 levels, which reduces the power consumption of the node when the weight data is not desired.

Using a rechargeable lithium-ion battery of 3.7 V/2300 mAh, a minimum autonomy of 75 h is achieved. This is enough time to solve the problem since the global server of the Wbee system generates an alarm when losing communication. Greater autonomy can be achieved by increasing the acquisition period.

The system is scalable regarding the number of nodes and physical-measurement parameters. The architecture used to implement the system allows the data of all colonies to be accessible on the Internet through one unique cloud database server. It is also possible to access the data of a beehive on the Internet by connecting to the local database server.

We have been able to prove the reliability of the WBee system without affecting the precision of the measurements, against the propolis-covering action of the bees and the environmental conditions of the area where the experiment was developed, in low temperatures in winter (especially during the night), and in high temperatures during the rest of the year, above all, in summer.

The system was installed beginning in September 2015, providing data to a vet team from the University of Córdoba of the AGR-218 Research Group (Improvement and Maintenance of Genetic Resources of Domestic Animals, Apiculture Unit) for the study of the relationships between the bee colonies and the environment and of their management by beekeepers. The analysis of the data has allowed us to check the ability of honeybees to regulate temperature and humidity under tested conditions, as well as how beekeepers handle the beehives affects those conditions and their relationship with the health of the hives and their production.

The measurement of the weight of the hives has allowed us to understand the evolution of the bee colonies during blooms, in particular, during a commercial sunflower bloom, as well as its practical application in usual management by beekeepers. As an example, it allows us to register the evolution of the production of honey in the hives or the end of the bloom and estimate the production of honey or indicate the most appropriate moment for the collection of the honey in advance, preventing unnecessary trips to the apiary by the beekeeper.

## Figures and Tables

**Table 1:** Power consumption according to manufacturer’s data.

Component	Operation Mode	Supply Current (Max)	Voltage
SHT15	Sleep	1.5 μA	5 VDC
	Measuring	1 mA	
	Average	28 μA	
Xbee Pro	idle/receive	55 mA	3.3 VDC
	Power down	<10 μA	
	Transmission	250 mA	
Waspmote	On	17 mA	3.3 VDC
	Deep sleep	33 μA	
	Hibernation	7 μA	

**Table 2:** Measured power consumption of the node.

Mode	Operation	Supply Current
Sleep	Sleep	45 µA
Active with communication	Measuring SHT15	70.5 mA
	Reading scale	70.2 mA
	Transmitting	271.1 mA
